# Anomalous origin of the left coronary artery from the pulmonary artery in infancy with preserved left ventricular function: Potential pitfalls and clues to diagnosis

**DOI:** 10.4103/0974-2069.41061

**Published:** 2008

**Authors:** Renu P. Kurup, Rachel Daniel, Raman Krishna Kumar

**Affiliations:** Department of Cardiology, Malabar Institute of Medical Sciences, Kozhikode, India; 1 Department of Cardiology, Kerala Institute of Medical Sciences, Trivandrum, India; 2 Department of Pediatric Cardiology, Amrita Institute of Medical Sciences and Research Center, Kochi, India

**Keywords:** Cardiac failure, left ventricular dysfunction, echocardiography, infants

## Abstract

Left ventricular dysfunction is almost invariably associated with anomalous origin of the left coronary artery from pulmonary artery (ALCAPA) that presents during infancy. We report three cases of infants who presented with ALCAPA with relatively well-preserved left ventricular systolic function with a view to illustrate the mechanisms that help maintain left coronary perfusion and discuss the specific echocardiographic clues that suggest diagnosis in these circumstances.

## INTRODUCTION

Anomalous origin of left coronary artery from pulmonary artery (ALCAPA) is one of the most frequently encountered congenital coronary abnormalities in children.[1] Typically, this condition manifests during infancy with heart failure resulting from reduced ventricular function often with mitral valve regurgitation that results from myocardial ischemia because of reduced perfusion in the territory supplied by the left coronary artery. This impaired perfusion is the result of declining pulmonary artery pressure secondary to a progressive decline in pulmonary vascular resistance.[2] The diagnosis of ALCAPA is usually considered in infants with otherwise unexplained left ventricular dysfunction or mitral valve regurgitation. Accurate diagnosis is often feasible through echocardiography. This involves demonstration of the origin of the left coronary artery from pulmonary artery together with reversed flow in the left coronary artery and its main divisions.[3,4] Occasionally, the disease escapes detection during infancy and is diagnosed much later in life. In these older patients the ventricular function tends to be preserved through extensive collateral supply from the right coronary artery branches.[5]

We describe three infants with ALCAPA in whom the left ventricular function was preserved. These three clinical examples illustrate mechanisms and associated conditions that allow preservation of left coronary artery flow. The echocardiographic clues to the diagnosis in presence of normal ventricular function will also be discussed.

## CASE REPORTS

### CASE 1

**Figure 1:**
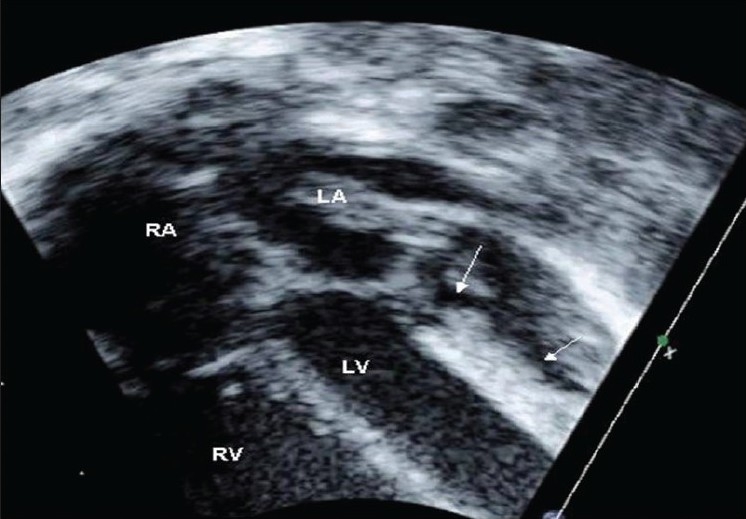
The left ventricle in case 1. Arrow marks showing hyperechogenic scarred papillary muscles. LA: Left Ventricle, LV: Left Ventricle, RA: Right Atrium, RV: Right Ventricle

**Figure 2:**
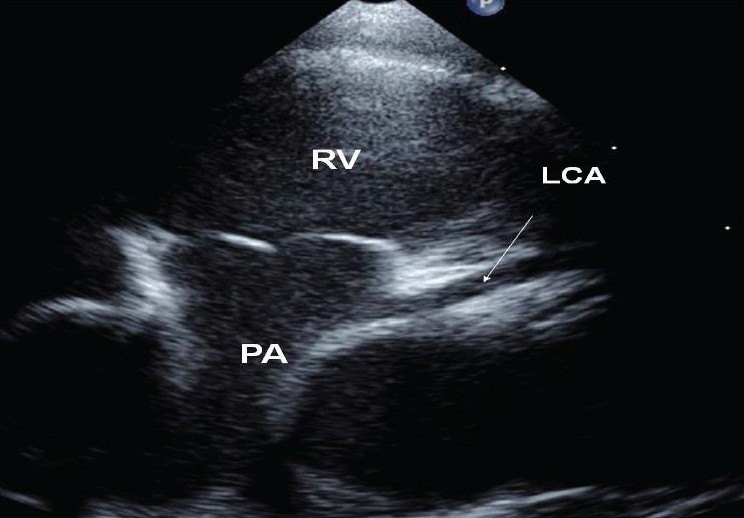
Modified parasternal view showing origin of left coronary artery from pulmonary artery. LCA: left coronary artery, PA: pulmonary artery, RV: right ventricle.

A 90-day-old girl was referred to our institution for respiratory distress and feeding difficulty. The chest X-ray showed mild cardiac enlargement. Electrocardiogram revealed sinus tachycardia, counterclockwise loop and a QRS axis of +150°. There were no q waves in lead avL and I or on the lateral chest leads. Echocardiogram at the initial presentation revealed a 9-millimeter atrial septal defect shunting left to right and normal ventricular function (ejection fraction 62%). The antero-lateral papillary muscle appeared bright in the echocardiogram. [Figure 1]. The coronary anatomy was initially thought to be normal with normal color Doppler flow. Medications to treat congestive heart failure were administered and close follow-up was advised. She was seen again at one month and the echocardiogram was repeated. On thorough interrogation of the coronaries, the left coronary artery was found to arise from the main pulmonary artery [Figure 2]. The direction of blood flow in the left coronary artery was away from the aorta. The right coronary artery was dilated and the abnormality of the papillary muscle persisted. The atrial septal defect was large and there was evidence of pulmonary artery hypertension with pressures nearly equal to systemic pressures. The left ventricular function was preserved. Surgical correction was advised.

The coronary artery was successfully translocated and the atrial septal defect was closed with 4 mm fenestration in view of the pulmonary hypertension. The child recovered uneventfully and was discharged on the 8^th^ post-operative day. Predischarge echocardiogram showed good biventricular function with minimal mitral regurgitation and normal pulmonary artery systolic pressures. The patient was well at her three month follow-up visit.

### CASE 2

A seven-month-old girl presented with history of interrupted feeds and failure to thrive since the second month of life. She was diagnosed to have mitral regurgitation at three months and was on medications to treat congestive heart failure (digoxin, furosemide and enalapril). Clinical examination revealed cardiac enlargement with a pan systolic murmur at the apex. Electrocardiogram showed sinus tachycardia with deep q waves in V6.

Echocardiogram revealed anomalous origin of left coronary artery from the pulmonary artery with no flow reversal in the left coronary artery and increased brightness of the antero-lateral papillary muscle and adjacent free wall. There was severe mitral regurgitation, severe pulmonary artery hypertension and normal left ventricular function (ejection fraction: 66%). The family declined surgery and the patient was lost to follow-up.

### CASE 3

**Figure 3:**
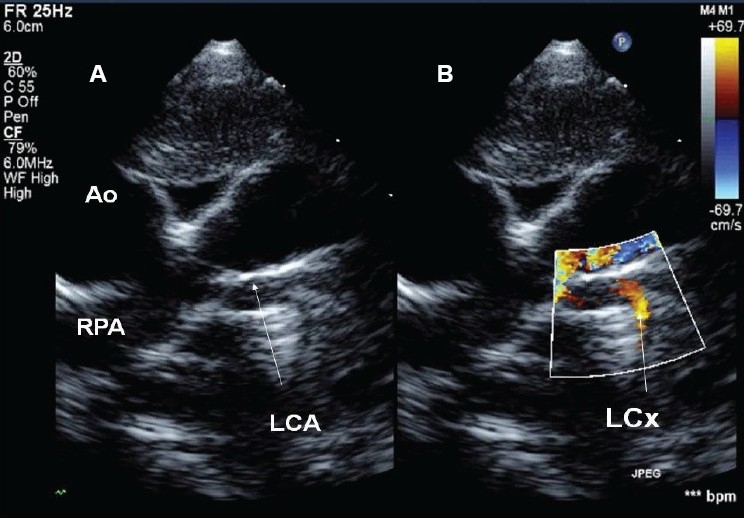
(A, B): These echocardiograms have been obtained from patient #3. (A) Left coronary artery arising from right pulmonary artery (B) retrograde flow from coronary to pulmonary artery. Ao: aorta, LCA: left coronary artery, LCx: Left circumflex artery, RPA: right pulmonary artery

A 10-month-old male infant was incidentally diagnosed to have tetralogy of Fallot at the age of 30 days. The child returned for surgery at the age of 10 months. He continued to remain pink and oxygen saturation was 98% prior to surgery. During the preoperative evaluation of coronaries, the right coronary artery was dilated. In addition, left coronary artery showed flow reversal [Figure 3]. On careful scanning the left coronary artery was found to arise from proximal right pulmonary artery. The left ventricular function was normal and the mitral valve papillary muscles were normal. The child underwent corrective operation with coronary translocation and recovered uneventfully (hospital stay of 9 days) and was doing well on follow-up evaluations at three months and one year.

## DISCUSSION

ALCAPA with preserved left ventricular function is well described in adults.[5] It is rare for an infant to have normal left ventricular function at presentation.[6] This report of three infants with anomalous origin of left coronary artery and preserved ventricular function is written to illustrate the circumstances where ventricular function may be preserved. In the absence of ventricular dysfunction this condition can be missed during initial echocardiography because it may not be suspected in the first place. Preserved ventricular function can result from preserved forward flow in the left coronary artery or rapid development of collateral flow from the right coronary artery.[5,7] Pulmonary hypertension allows forward flow in the left coronary artery in patients with anomalous origin of left coronary artery.[6,8,9]

In the first patient, high pulmonary artery pressures were consistently seen. This patient had an atrial septal defect and would normally have had a decline in pulmonary arterial pressures at the time of presentation. However she continued to have severe pulmonary hypertension and forward flow in the left coronary artery. Here, the clue to the diagnosis of anomalous left coronary artery was the increased echogenecity of the anterior-lateral papillary muscles in comparison to the left ventricular free wall and ventricular septum. It is possible that the reduced left ventricular compliance may have substantially increased the shunt across the atrial septal defect. The likely explanation for preserved ventricular function in the second patient was pulmonary hypertension resulting from severe mitral valve regurgitation. This patient also had increased echogenecity of anterior-lateral papillary muscle. The third patient had tetralogy of Fallot and therefore pulmonary artery pressures were low. We speculate that this patient rapidly developed a good collateral supply from the right coronary artery. The association of anomalous origin of left coronary artery from pulmonary artery with tetralogy of Fallot and preserved ventricular function has been reported previously.[10] Collaterals flow from right coronary artery into the pulmonary artery may also have helped in maintaining good oxygen saturations.

The classical echocardiographic finding in the diagnosis of anomalous left coronary artery is demonstration of the ALCAPA by two-dimensional echocardiography.[3,4] Demonstration of color Doppler flow reversal in proximal left coronary artery is considered crucial because errors may result if two-dimensional echocardiographic images are used in isolation.[4] While pulmonary hypertension allows preservation of ventricular function, it may make echocardiographic diagnosis difficult. The increased echogenecity of anterior-lateral papillary muscle and adjacent myocardium on echocardiography is a useful pointer to the diagnosis in this situation.[3] The presence of this finding should alert the echocardiographer to carefully look at the origin of the left coronary artery.

It is important to recognize that there are clinical clues that point to the diagnosis of ALCAPA. The earliest symptoms include inconsolability and feeding difficulties. Often there are no obvious abnormal cardiovascular findings at this stage and pediatricians may have difficulty in ascribing these symptoms to ALCAPA because a number of relatively common infantile ailments would first be considered. These early symptoms were not appreciated in case 1 and perhaps in case 2 as well. When these clues are present, the diagnosis of ALCAPA should be considered and careful echocardiographic interrogation of the coronary artery origins and flow is warranted.

In conclusion, left ventricular function can occasionally be preserved in infants with ALCAPA. Attention to specific echocardiographic clues such as increased echogenecity of the left ventricular papillary muscle, excessive dilation of right coronary artery can minimize the chances of missing the diagnosis of this potentially life-threatening condition.
